# Need for formalized robotic training and curriculum in obstetrics and gynecology residency: an examination of current resident outlooks and perspectives

**DOI:** 10.1007/s11701-024-01985-9

**Published:** 2024-05-21

**Authors:** Anusha Adkoli, Samanatha Eng, Ruth Stephenson

**Affiliations:** 1 Rutgers Robert Wood Johnson Medical School, 125 Paterson St, New Brunswick, NJ 08901 USA; 2 Rutgers Cancer Institute of New Jersey, New Brunswick, NJ 08901 USA

**Keywords:** Education, Residency, Robotics, Survey, Training

## Abstract

The objectives of this study were to evaluate current robotic surgery training methodologies for ACGME-accredited obstetrics and gynecology (OB/GYN) residency programs, better understand current resident perspectives, and explore potential areas for improvement within resident education. A cross-sectional study was done of ACGME-accredited OB/GYN residents in the 2023–2024 academic year. The study was done on a national setting via web-based survey. 75 surveys were included. The study was conducted via a 33-question survey study using a mixture of multiple choice, multiple answer, and Likert scale questions. Participants noted that 98.7% of their institutions perform robotic surgery and 90.7% have access to robotic console trainers. Outside of the operating room, slightly more than half of participants (57.3%) have formalized robotics training curriculums. A variety of training modalities were noted to be utilized by residents with the most helpful being hands-on training (67.7%) followed by dual-assist console (45.6%). The least helpful was noted to be online modules (58.7%). Most residents either strongly agree (45.3%) or agree (36.0%) that standardized robotics curriculums should be implemented for all OB/GYN residency programs. The largest barriers to completion of this training were noted to be attending comfort with resident participation in the case (74.0%), personal time (58.9%), and availability or access to trainers (42.5%). A formalized and standardized robotic training curriculum should be considered for OB/GYN residents with a multi-modal model utilizing a combination of training modalities as well as dedicated didactic hours.

## Introduction

Robotic-assisted surgery (RAS) allows for surgeons to operate in a minimally invasive approach while having increased dexterity and range of motion due to ability to articulate wrist and hand motions [1]. Other reported advantages to robotic surgery include improved ergonomics, elimination of tremor, and improved visualization [2]. Over 11 million robotic surgeries have been performed worldwide with over 7500 platforms installed for use as of 2023 [3].

To be able to perform robotic surgeries proper training is required, but current guidelines for credentialing and privileges vary across institutions and hospitals [2]. Current guidelines exist from commercial robotics companies; however, there are no standardized curriculums for robotic training during obstetrics and gynecology residency (OB/GYN) despite the robust use of robotics systems. The American College of Obstetricians and Gynecologists (ACOG) and Society of General Surgeons (SGS) both recommend that those training requirements to include a didactic educational program as well as hands-on training which would include bedside assisting and sitting on the console [2].

To evaluate resident perspectives on robotics training, a nationwide survey of OB/GYN residents was done in 2015–2016 which found that 35% of participants did not have access to any robotics training and the greatest barriers to education were time and proper equipment [4]. Compared to a similar prior 2011 survey, there was an increase in completion of robotics training programs in 65% of those interviewed vs 16% [5]. Since then there has been a push to implement a more formalized curriculum for robotic-assisted surgery within OB/GYN residency [5]. It is known that there has been a substantial increase in the number of robotic-assisted surgeries since 2015. However, it is not well studied how resident surgical training curriculums and perspective have changed over the years with this continued rise.

The motivation of this study is to evaluate if ACGME-accredited OB/GYN residency programs have evolved with the changing landscape of gynecologic surgery. The objectives of this study were to evaluate current robotic surgery training methodologies for ACGME-accredited OB/GYN residency programs, better understand current resident perspectives, and explore potential areas for improvement within resident education.

## Methods

This study was an IRB-approved multi-institutional survey of ACGME-accredited OB/GYN residents enrolled in the 2023–2024 academic year. A 33-question survey study was created with a mixture of multiple choice, multiple answer, and Likert scale questions to evaluate resident access, education, and outlooks on robotic surgery training during residency. The survey was developed utilizing templates from similar prior studies. The survey was distributed via Survey Monkey platform using their anonymous collection platform. There are currently 292 OB/GYN programs in the United States. The survey was sent via email to all accredited OB/GYN program coordinators to then forward to their residents for completion. No incentives were offered for completion. At the beginning of the survey, a brief description regarding the survey was provided. The participant then had the option to continue with the survey as a means of consent to the survey. Alternatively, participants could exit and choose not to complete the survey. Respondents could review and change their responses before the final submission. Responses were collected over the course of 4 months during which time email reminders were sent periodically to program coordinators to forward to their residents. Responses were automatically collected into the survey website which was password protected. Data were downloaded onto secure password protected computers. Exclusion criteria included those who have already completed a prior surgical residency prior to OB/GYN and any OB/GYN faculty other than residents. Given that participants are residents within ACGME-accredited programs, participants were assumed to be over the age of 18 and able to read English. Results were analyzed primarily in a descriptive manner with a percentage breakdown per response.

## Results

### Demographics

There were 75 surveys completed. Although the number of OB/GYN residents nationally can be determined, this is not reflective of the true sample size. The true sample size is unknown given due to the method of distribution. It is unable to be determined how many residents received the survey due to the nature of the anonymity of the survey. Surveys with missing data were not excluded due to the nature of the survey questions and possibility of certain questions being inapplicable to the participant. The number of participants is reported for each question. Most of the participants were white (67.6%), female (91.9%) and between age 26 and 30 (64.0%). Majority were post-graduate year (PGY) 4 (40.0%) or PGY3 (32.0%) from an academic institution (74.7%). Geographically, most participants attended programs in the Northeast (52.0%) followed by Southeast (18.7%) and West (14.7%) (Table 1).

**Table 1:** Demographics

Demographic variable	Percentage	Frequency
*Age* (*n* = 75)		
20–25	1.3%	1
26–30	64.0%	48
31–35	32.0%	24
36–40	2.7%	2
*Gender* (*n* = 74)		
Female	91.9%	68
Male	8.1%	6
Gender neutral	0.0%	0
Non-binary	0.0%	0
*Race* (*n* = 74)		
White	67.6%	50
Black	9.5%	7
Asian	14.9%	11
Hispanic	4.0%	3
Other	4.0%	3
*Post-graduate year* (*n* = 75)		
PGY1	9.3%	7
PGY2	18.7%	14
PGY3	32.0%	24
PGY4	40.0%	30
*Type of program* (*n* = 75)		
Community	25.3%	19
Academic	74.7%	56
*Geographical region* (*n* = 75)		
Northeast	52.0%	39
Southwest	2.7%	2
West	14.7%	11
Southeast	18.7%	14
Midwest	9.3%	7

Note: *Other: write in answers = Arab, biracial, prefer not to answer

### Robotics surgical exposure

Most institutions (98.7%) perform robotic surgeries with most residents getting involved in cases at the PGY1 (45.3%) or PGY2 (46.7%) level. Involvement via bedside assistance for most participants started at as a PGY1 (45.3%) or PGY2 (40.0%). Most residents can sit on the robotic console during surgery (93.3%) which primarily starts as a PGY3 (46.7%) or PGY2 (32%). However, most residents have never been the primary surgeon (60%), defined by completion of at least half the case. The rotations during which most residents were exposed to robotic surgery included gynecologic oncology (94.6%) followed by benign gynecology (83.8%) and urogynecology/female pelvic medicine and reconstructive surgery (66.2%) (Table 2).

**Table 2:** Operating room experience

Operating room experience	Percentage	Frequency
*Does your institution perform robotic surgeries*? (*n* = 75)		
Yes	98.7%	74
No	1.3%	1
*At what PGY year do you start getting involved in robotic surgery*? (*n* = 75)		
PGY1	45.3%	34
PGY2	40.0%	30
PGY3	12.0%	9
PGY4	2.7%	2
*At what PGY year do you bedside assist in robotic surgery*? (*n* = 75)		
PGY1	45.3%	34
PGY2	46.7%	35
PGY3	6.7%	5
PGY4	1.33%	1
*Does your program allow residents to sit on the console*? (*n* = 75)		
Yes	93.3%	70
No	6.7%	5
*At what PGY year do you sit on the console in robotic surgery*? (*n* = 75)		
PGY1	9.3%	7
PGY2	32.0%	24
PGY3	46.7%	35
PGY4	6.7%	5
*How many robotic cases have you participated in until this point as a resident*? (*n* = 75)		
0 cases	9.3%	7
1–5 cases	21.3%	16
6 -9 cases	14.7%	11
10 20 cases	16.0%	12
21 or more	38.7%	29
*Have you ever been the primary surgeon for a robotic case, if yes how many cases*? (*n* = 75)		
No	60.0%	45
Yes, 0–1 case	5.3%	4
Yes 2–5 cases	8.0%	6
Yes 6–10 cases	12.0%	9
Yes > 11 cases	14.7%	11
*During which clinical rotations do you get exposure to robotic surgery*? (*n* = 74)		
Benign gynecology	83.8%	62
Gynecologic oncology	94.6%	70
*Urogynecology*/*pelvic*		
Floor reconstruction	66.2%	49
*Minimally invasive*		
Gynecologic surgery	4.0%	3

### Robotics simulation and education

**Fig. 1:**
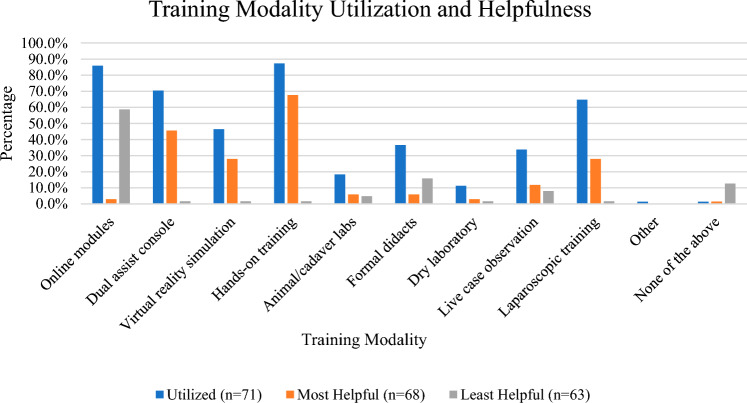
Training modality utilization and helpfulness

Outside of the operating room, slightly more than half of participants (57.3%) have formalized robotics training curriculums. Required didactics training taught by faculty is noted to be nonexistent or minimal at 0 h (56.7%) or 1–2 h (20.9%). In terms of simulation training, most residents noted access to a robotic console trainer (90.7%), while less residents noted to have easy access to training simulators as a whole (72.0%). Completion of virtual reality simulation modules is not required as part of robotic training for most residents (62.5%). Those who did complete the modules noted spending between 3-5 h (36.4%) or 6–10 h (25%) completing them (Table 3; Fig. 1).

**Table 3:** Training simulation and robotic education

Training simulation and robotic education	Percentage	Frequency
*Do you have a formalized robotic training curriculum*? (*n* = 75)		
Yes	57.3%	43
No	42.7%	32
*How many hours of robotic didactic training taught by faculty are required at your program*? (*n* = 67)		
0 h	56.7%	38
1–2 h	20.9%	14
3–5 h	13.4%	9
6–10 h	4.5%	3
11 h or more	4.5%	3
*Do you have access to a robot console trainer*? (*n* = 75)		
Yes	90.7%	68
No	9.3%	7
*Do you have easy access to training simulators*? (*n* = 75)		
Yes	72.0%	54
No	28.0%	21
*Is completing virtual reality simulation modules (da Vinci Skill Simulator) a required part of your residency training*? (*n* = 72)		
Yes	37.5%	27
No	62.5%	45
*If you completed virtual reality simulation modules (I.e., da Vinci Skill Simulator) how many hours did it take to complete*? (*n* = 44)		
0 h	15.9%	7
1–2 h	9.1%	4
3–5 h	36.4%	16
6–10 h	25.0%	11
11 h or more	13.6%	6
*Are you robot certified*? (*n* = 75)		
Yes	9.3%	7
No	90.7%	68
*Do you plan to aim to become robot certified by the time of graduation from residency*? (*n* = 74)		
Yes	79.7%	59
No	20.3%	15
*What are the biggest barriers to completion of robotic training? Select all that apply* (*n* = 73)		
None of the above	2.7%	2
Personal time to complete requirements	58.9%	43
Availability or access to robot/simulator	42.5%	31
Fellow involvement in surgical cases	20.6%	15
Number of residents requiring training	8.2%	6
Attending comfort with resident participation in robotic cases	74.0%	54
Other (please specify) *	9.6%	7

^*^Other = amount of benign GYN surgeons that use the robot, no guidance, and limited number of attendings doing cases

A variety of training modalities were noted to be utilized by residents for robotic surgery training including online modules (85.9%), dual-assist console (70.4%), virtual reality simulation (46.5%), hands-on training (87.3%), animal/cadaver labs (18.3%), formal didactics (36.6%), dry laboratory (11.3%), live case observation (64.8%), laparoscopic training (64.8%), and other (1.41%). The training modality written in under other was E-MIGS. Of these utilized modalities, the methods that residents found most helpful were hands-on training (67.7%) followed by dual-assist console (45.6%). The least helpful was noted to be online modules (58.7%).

At the time of the survey, most residents were not robotic certified (90.7%) but were planning to achieve certification by the time of graduation (79.7%). The largest barriers to completion of this training were noted to be attending comfort with resident participation in the case (74.0%), personal time (58.9%), and availability or access to trainers (42.5%).

### Resident perspectives and future directions

**Fig. 2:**
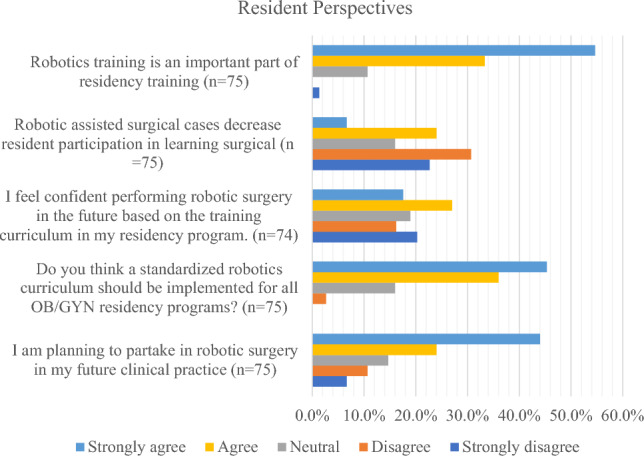
Resident perspectives

**Fig. 3:**
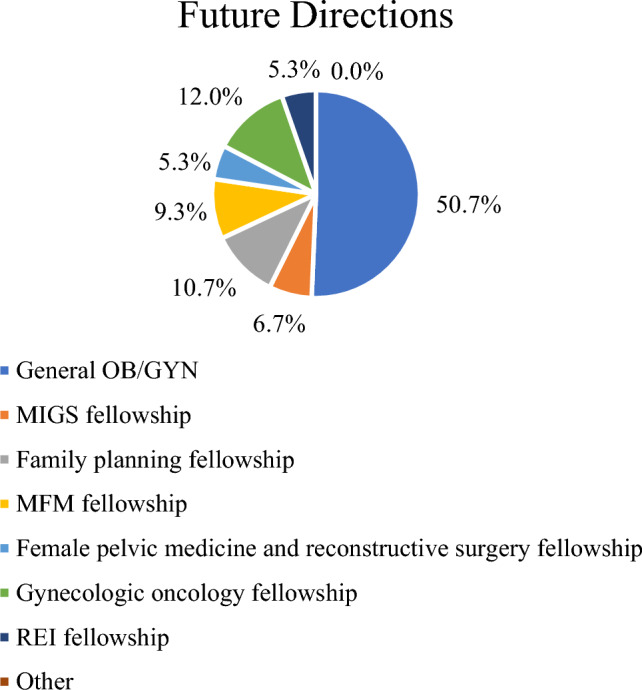
Future directions

From a resident perspective, most agree (33.3%) or strongly agree (54.7%) that robotics training is an important part of residency training and do not believe that a rise in robotics cases detract from resident learning (53.3%). There is a wide variation in confidence of future skills with most residents agreeing (27.0%) or strongly agreeing (17.6%) that they feel confident performing robotic surgery based on their training, while another large subset either strongly disagreed (20.3%) or disagreed (16.2%) with that statement (Figs. 2, 3).

For future careers paths, most strongly agree (44.0%) or agree (24%) that they will be using robotic surgery in future clinical practice. The distribution of future career paths was about half generalists (50.7%) and the other a mixture of various subspecialties including gynecologic oncology (12.0%), family planning (10.7%), and maternal fetal medicine (9.3%) (Fig. 3).

Importantly, most residents either strongly agree (45.3%) or agree (36.0%) that standardized robotics curriculums should be implemented for all OB/GYN residency programs. Another subset felt neutral (16.0%) regarding that statement and only a small minority disagreed (2.7%).

## Discussion

In 2014, the number of robotic-assisted surgeries (RAS) was over 570,000 in the Unites States alone [4]. In 2020, this number was 876,000 despite decrease in elective cases due to COVID-19 [6]. Furthermore, an analysis by Michigan Surgical Quality Collaborative registry showed a rise between 2012 and 2018 of 1.8% to 15.1% for robotic cases [7].

The FDA approved RAS for gynecologic surgery in 2005 with rapid growth of use since then [2]. Although commonly used for general surgery procedures, according to Intuitive Surgical 2021 report, gynecology was the second largest surgical subspecialty using robotic systems in the United States [8]. A parallel increase has been seen with a rise in robotic hysterectomies with a decline in abdominal, laparoscopic, and vaginal hysterectomies [8]. Numbers have also risen within subspecialities of gynecology such as gynecologic oncology with a rise in minimally invasive staging procedures for uterine cancer after the LAP2 trial illustrated it as a feasible and safe alternative to open surgical staging [9].

Over the years, similar surveys to this have been performed to assess resident perspectives on robotic surgery training, education, and curriculums. These studies showed an increase in access to robotic consoles at training institutions from 58% to 98.9% between 2011 and 2014 [4]. This number has remained consistent in this survey with 98.7% of institutions performing robotic surgery and 90.7% having access to robotic console trainers.

Similar to prior studies, time was a major barrier to completion of robotics training for 58.9% of respondents. Interestingly, an even larger barrier for 74.0% of residents was noted to be attending comfort with resident participation in robotic surgeries. This is further demonstrated in that residents have participated in both bedside assisting and sitting on the console; however, 60% have never been primary surgeon, meaning completing at least half the case. This barrier may reflect the curriculum residents are receiving with robotics. Only 57.3% of residents are receiving any sort of formalized curriculum, which may translate to attending lack of comfort with allowing residents as primary surgeon. Based on our survey, this firsthand training was found to be most helpful in training.

After the 2015 study, there was a push for creation of standardized curriculums for robotic surgery within OB/GYN residency [5]. Potential standardized and certified training pathways are being explored on a global level through utilization of web-based training, simulation, on-site training, and mentorship [10]. A U.K.-based survey study further illustrated OB/GYN trainee interest in receiving formal education for first assist and console role [11]. The study utilized a Delphi technique with four rounds of questions to illustrate that it is possible to come to a consensus on the core curriculum for this training [11].

In terms of future directions, approximately 80% of OB/GYN residents in our study are aiming to become robot certified and 68% are planning to continue use of robotics in future clinical practice. In addition to the absence of standardized training programs, robotics certification itself also varies by institution with a lack of criteria and benchmarks based on ACGME requirements [2]. Although other areas of surgery within gynecology such as vaginal surgery or laparoscopy do not have standardized curriculums nationally, there are a certain number required for graduation creating some level of standardization nationally. Current requirements for laparoscopic cases for graduation from OB/GYN residency include 60 laparoscopy cases, 15 of which are hysterectomies, but none of which are required to be robotic cases [12].

Furthermore, hospital requirements for privileges with robotic surgery post-graduation also vary greatly. The American Associated of Gynecologic Laparoscopists have suggested guidelines regarding privileging including pre-requisites such as performing a minimum of 20 robotic cases per year [13]. The premise is that the surgeon must have the judgment and training to complete the procedure safely as intended and institutions require previous case logs as primary surgeon as well as proctoring based on medical staff bylaws to perform robotic surgery. If most OB/GYN residents are aiming to use robotics in their future practice, the required training and experience is needed to obtain privileges as an attending surgeon.

Our survey study illustrates this need for a formalized curriculum and benchmarks for robotic surgery in OB/GYN residency, limitations of this study include the low response rate. Additionally, given the method of distribution, we cannot determined how many residents received the survey to complete. However, the distribution of geographic locations and years of residents indicate that it was distributed nationally and across post-graduate levels. Future survey studies may benefit from using a larger scale platform, for example through the American College of Obstetrics and Gynecology, or incentivization for survey completion. Another limitation for consideration is that the topic of robotic surgery may appeal to residents based on interest in the field, which may create a bias in those who complete the survey. However, based on the responses received, most residents think there should be a standardized curriculum implemented, even if there is a variation of interest in robotic surgery itself.

In 2015, 42.5% of residents had said that they strongly agree or agree with the statement that they would use robotics in their future practice [4]. This is compared to 68.0% in our survey, illustrating a parallel between rising rates of robotic gynecologic surgery and resident plans for future practice in robotic surgery. For this reason, a formalized and standardized robotic training curriculum that allows trainees to obtain the necessary cases and primary surgeon experience is necessary for OB/GYN residency training. This would allow residents to gain necessary skills in a timely manner and competency for future careers in robotic gynecologic surgery.

## Data Availability

No datasets were generated or analysed during the current study.

## Declarations

### Prior presentation

Has not been presented prior.
